# Existence Results for a System of Coupled Hybrid Fractional Differential Equations

**DOI:** 10.1155/2014/426438

**Published:** 2014-08-18

**Authors:** Bashir Ahmad, Sotiris K. Ntouyas, Ahmed Alsaedi

**Affiliations:** ^1^Nonlinear Analysis and Applied Mathematics (NAAM) Research Group, Department of Mathematics, Faculty of Science, King Abdulaziz University, P.O. Box 80203, Jeddah 21589, Saudi Arabia; ^2^Department of Mathematics, University of Ioannina, 451 10 Ioannina, Greece

## Abstract

This paper studies the existence of solutions for a system of coupled hybrid fractional differential equations with Dirichlet boundary conditions. We make use of the standard tools of the fixed point theory to establish the main results. The existence and uniqueness result is elaborated with the aid of an example.

## 1. Introduction

Fractional calculus is the study of theory and applications of integrals and derivatives of an arbitrary (noninteger) order. This branch of mathematical analysis, extensively investigated in the recent years, has emerged as an effective and powerful tool for the mathematical modeling of several engineering and scientific phenomena. One of the key factors for the popularity of the subject is the nonlocal nature of fractional-order operators. Due to this reason, fractional-order operators are used for describing the hereditary properties of many materials and processes. It clearly reflects from the related literature that the focus of investigation has shifted from classical integer-order models to fractional-order models. For applications in applied and biomedical sciences and engineering, we refer the reader to the books [1–4]. For some recent work on the topic, see [5–25] and the references therein. The study of coupled systems of fractional-order differential equations is quite important as such systems appear in a variety of problems of applied nature, especially in biosciences. For details and examples, the reader is referred to the papers [26–33] and the references cited therein.

Hybrid fractional differential equations have also been studied by several researchers. This class of equations involves the fractional derivative of an unknown function hybrid with the nonlinearity depending on it. Some recent results on hybrid differential equations can be found in a series of papers (see [34–37]).

Motivated by some recent studies on hybrid fractional differential equations, we consider the following Dirichlet boundary value problem of coupled hybrid fractional differential equations:

(1) Dcδ(x(t)f1(t,x(t),y(t)))=h1(t,x(t),y(t)),0<t<1, 1<δ≤2,Dcω(y(t)f2(t,x(t),y(t)))=h2(t,x(t),y(t)),0<t<1, 1<ω≤2,x(0)=x(1)=0,  y(0)=y(1)=0,

where ^*c*^ *D* ^*δ*^,  ^*c*^ *D* ^*ω*^ denote the Caputo fractional derivative of orders *δ*, *ω*, respectively, *f*_*i*_ ∈ *C*([0,1] × *R* × *R*, *R*∖{0}) and *h*_*i*_ ∈ *C*([0,1] × *R* × *R*, *R*), *i* = 1,2.

The aim of this paper is to obtain some existence results for the given problem. Our first theorem describes the uniqueness of solutions for the problem (1) by means of Banach's fixed point theorem. In the second theorem, we apply Leray-Schauder's alternative criterion to show the existence of solutions for the given problem. The paper is organized as follows.  Section 2 contains some basic concepts and an auxiliary lemma, an important result for establishing our main results. In Section 3, we present the main results.

## 2. Preliminaries

In this section, some basic definitions on fractional calculus and an auxiliary lemma are presented [1, 2].

**Definition 1 .**

The Riemann-Liouville fractional integral of order *q* for a continuous function *g* is defined as

(2) $$\begin{array}{c} I^{q}g\left(t\right)=\frac{1}{\Gamma \left(q\right)}\overset{t}{\underset{0}{\int }}\frac{g\left(s\right)}{{\left(t-s\right)}^{1-q}}ds,\;q>0, \end{array}$$

provided that the integral exists.

**Definition 2 .**

For at least *n*-times continuously differentiable function *g* : [0, *∞*) → *R*, the Caputo derivative of fractional-order *q* is defined as

(3) cDqg(t)=1Γ(n−q)∫0t(t−s)n−q−1g(n)(s)ds,n−1<q<n, n=[q]+1,

where [*q*] denotes the integer part of the real number *q*.

**Lemma 3 (auxiliary lemma).**

Given *ϕ* ∈ *C*([0,1], *R*), the integral solution of the problem

(4) $$\begin{array}{c} D^{\delta }\left(\frac{x\left(t\right)}{f\left(t,x\left(t\right),y\left(t\right)\right)}\right)=\phi \left(t\right),\;0<t<1,\\ x\left(0\right)=x\left(1\right)=0 \end{array}$$

is

(5) x(t)=f(t,x(t),y(t)) ×(∫0t(t−s)δ−1Γ(δ)ϕ(s)ds−t∫01(1−s)δ−1Γ(δ)ϕ(s)ds),t∈[0,1].

**Proof**

It is well known that the general solution of the fractional differential equation in (4) can be written as

(6) $$\begin{array}{c} \frac{x\left(t\right)}{f\left(t,x\left(t\right),y\left(t\right)\right)}=\overset{t}{\underset{0}{\int }}\frac{{\left(t-s\right)}^{\delta -1}}{\Gamma \left(\delta \right)}\phi \left(s\right)ds+c_{0}t+c_{1}, \end{array}$$

where *c*_0_, *c*_1_ ∈ *R* are arbitrary constants. Alternatively, we have

(7) x(t)=f(t,x(t),y(t)) ×(∫0t(t−s)δ−1Γ(δ)ϕ(s)ds+c0t+c1), t∈[0,1].

Using the given boundary conditions *x*(0) = 0 = *x*(1) in (7), we find that

(8) $$\begin{array}{c} c_{1}=0,\;\;c_{0}=-\overset{1}{\underset{0}{\int }}\frac{{\left(1-s\right)}^{\delta -1}}{\Gamma \left(\delta \right)}\phi \left(s\right)ds. \end{array}$$

Substituting the values of *c*_0_, *c*_1_ in (7) yields the solution

(9) x(t)=f(t,x(t),y(t)) ×(∫0t(t−s)δ−1Γ(δ)ϕ(s)ds−t∫01(1−s)δ−1Γ(δ)ϕ(s)ds),t∈[0,1].

This completes the proof.

## 3. Main Results

Let *W* = {*w*(*t*)∣*w*(*t*) ∈ *C* ^1^([0,1])} denote a Banach space equipped with the norm ||*w*|| = max⁡⁡{|*w*(*t*)|, *t* ∈ [0,1]}, where *W* = *U*, *V*. Notice that the product space (*U* × *V*, ||(*x*, *y*)||) with the norm ||(*x*, *y*)|| = ||*x*|| + ||*y*||, (*x*, *y*) ∈ *U* × *V* is also a Banach space.

In view of Lemma 3, we define an operator Θ : *U* × *V* → *U* × *V* by

(10) $$\begin{array}{c} \Theta \left(x,y\right)\left(t\right)=\left(\begin{array}{c} \Theta_{1}\left(x,y\right)\left(t\right)\\ \Theta_{2}\left(x,y\right)\left(t\right) \end{array}\right), \end{array}$$

where

(11) Θ1(x,y)(t)=f1(t,x(t),y(t)) ×(∫0t(t−s)δ−1Γ(δ)h1(s,x(s),y(s))ds −t∫01(1−s)δ−1Γ(δ)h1(s,x(s),y(s))ds),Θ2(x,y)(t)=f2(t,x(t),y(t)) ×(∫0t(t−s)ω−1Γ(ω)h2(s,x(s),y(s))ds −t∫01(1−s)ω−1Γ(ω)h2(s,x(s),y(s))ds).

In the sequel, we need the following assumptions.

*(*A*_1_*) The functions *f*_*i*_  (*i* = 1,2) are continuous and bounded; that is, there exist positive numbers *μ*_*f*_*i*__ such that |*f*_*i*_(*t*, *u*, *v*)| ≤ *μ*_*f*_*i*__, ∀(*t*, *x*, *y*) ∈ [0,1] × *R* × *R*.
( *A*_2_ ) There exist real constants *ρ*_0_, *σ*_0_ > 0 and *ρ*_*i*_, *σ*_*i*_ ≥ 0  (*i* = 1,2) such that |*h*_1_(*t*, *x*, *y*)| ≤ *ρ*_0_ + *ρ*_1_|*x*| + *ρ*_2_|*y*| and |*h*_2_(*t*, *x*, *y*)| ≤ *σ*_0_ + *σ*_1_|*x*| + *σ*_2_|*y*|, ∀*x*, *y* ∈ *R*, *i* = 1,2.

For brevity, let us set

(12) ν1=2μf1Γ(δ+1),  ν2=2μf2Γ(ω+1),

(13) ν0=min⁡⁡{1−(ν1ρ1+ν2σ1),1−(ν1ρ2+ν2σ2)}, ρi,σi≥0 (i=1,2).

Now we are in a position to present our first result that deals with the existence and uniqueness of solutions for the problem (1). This result is based on Banach's contraction mapping principle.

**Theorem 4 .**

Suppose that condition (*A*_1_) holds and that *h*_1_, *h*_2_ : [0,1] × *R* ^2^ → *R* are continuous functions. In addition, there exist positive constants *η*_*i*_, *ζ*_*i*_, *i* = 1,2 such that

(14) |h1(t,x1,y1)−h1(t,x2,y2)|≤η1|x1−x2|+η2|y1−y2|,|h2(t,x1,y1)−h2(t,x2,y2)|≤ζ1|x1−x2|+ζ2|y1−y2|,∀t∈[0,1], x1,x2,y1,y2∈R.

If *ν*_1_(*η*_1_ + *η*_2_) + *ν*_2_(*ζ*_1_ + *ζ*_2_) < 1*ν*_1_ and *ν*_2_ are given by (12), then the problem (1) has a unique solution.

**Proof**

Let us set sup⁡_*t*∈[0,1]_ *h*_1_(*t*, 0,0) = *κ*_1_ < *∞*, sup⁡_*t*∈[0,1]_ *h*_2_(*t*, 0,0) = *κ*_2_ < *∞* and define a closed ball: ${\overset{-}{B}}_{r}=\left\{\left(x,y\right)\in U\times V:||\left(x,y\right)||\leq r\right\}$, where

(15) $$\begin{array}{c} r\geq \frac{\kappa_{1}\nu_{1}+\kappa_{2}\nu_{2}}{1-\nu_{1}\left(\eta_{1}+\eta_{2}\right)-\nu_{2}\left(\zeta_{1}+\zeta_{2}\right)}. \end{array}$$

Then we show that $\Theta {\overset{-}{B}}_{r}\subset {\overset{-}{B}}_{r}$. For $\left(x,y\right)\in {\overset{-}{B}}_{r}$, we obtain

(16) |Θ1(x,y)(t)| ≤Mf1max⁡t∈[0,1]⁡{∫0t(t−s)δ−1Γ(δ)|h1(s,x(s),y(s))|ds  +∫01(1−s)δ−1Γ(δ)|h1(s,x(s),y(s))|ds} ≤Mf1max⁡t∈[0,1]⁡{∫0t(t−s)δ−1Γ(δ)  ×(|h1(s,x(s),y(s))−h1(s,0,0)|  +|h1(s,0,0)|)ds+∫01(1−s)δ−1Γ(δ)  ×(|h1(s,x(s),y(s))−h1(s,0,0)|  +|h1(s,0,0)|)ds} ≤2Mf1Γ(δ+1)(η1||x||+η2||y||+κ1) ≤ν1[(η1+η2)r+κ1].

Hence

(17) $$\begin{array}{c} ||\Theta_{1}\left(x,y\right)||\leq \nu_{1}\left[\left(\eta_{1}+\eta_{2}\right)r+\kappa_{1}\right]. \end{array}$$

Working in a similar manner, one can find that

(18) $$\begin{array}{c} ||\Theta_{2}\left(x,y\right)||\leq \nu_{2}\left[\left(\zeta_{1}+\zeta_{2}\right)r+\kappa_{2}\right]. \end{array}$$

From (17) and (18), it follows that ||Θ(*x*, *y*)|| ≤ *r*.

Next, for (*x*_1_, *y*_1_), (*x*_2_, *y*_2_) ∈ *U* × *V* and for any *t* ∈ [0,1], we have

(19) |Θ1(x2,y2)(t)−Θ1(x1,y1)(t)| ≤μf1∫0t(t−s)δ−1Γ(δ)|h1(s,x2(s),y2(s))  −f(s,x1(s),y1(s))|ds  +∫01(1−s)δ−1Γ(δ)|h1(s,x2(s),y2(s))  −f(s,x1(s),y1(s))|ds ≤2μf1Γ(δ+1)(m1|u2−u1|+m2|v2−v1|) ≤ν1(η1||x2−x1||+η2||y2−y1||) ≤ν1(m1+m2)(||x2−x1||+||y2−y1||),

which yields

(20) ||Θ1(x2,y2)(t)−Θ1(x1,y1)|| ≤ν1(m1+m2)(||x2−x1||+||y2−y1||).

Similarly, one can get

(21) ||Θ2(x2,y2)(t)−Θ2(x1,y1)|| ≤ν2(ζ1+ζ2)(||x2−x1||+||y2−y1||).

From (20) and (21), we deduce that

(22) ||Θ(x2,y2)−Θ(x1,y1)||≤[ν1(η1+η2)+ν2(ζ1+ζ2)] ×(||x2−x1||+||y2−y1||).

In view of condition *ν*_1_(*η*_1_ + *η*_2_) + *ν*_2_(*ζ*_1_ + *ζ*_2_) < 1, it follows that Θ is a contraction. So Banach's fixed point theorem applies and hence the operator Θ has a unique fixed point. This, in turn, implies that the problem (1) has a unique solution on [0,1]. This completes the proof.

**Example 5 .**

Consider the following coupled system of hybrid fractional differential equations:

(23) Dc3/2(u(t)(1/2)(|sinu(t)|+1))=14(t+2)2|u(t)|1+|u(t)|+1   +132sin2v(t), t∈[0,1],Dc3/2(v(t)(1/2)(|cos⁡⁡u(t)|+1))=132πsin⁡(2πu(t))   +|v(t)|16(1+|v(t)|)+12, t∈[0,1],u(0)=0,  u(1)=0,v(0)=0,  v(1)=0.

Here *δ* = 3/2, *ω* = 3/2, *f*_1_(*t*, *u*, *v*) = (1/2)(|sin⁡*u*(*t*)| + 1), *f*_2_(*t*, *u*, *v*) = (1/2)(|cos⁡⁡*u*(*t*)| + 1), *h*_1_(*t*, *x*, *y*) = (1/4(*t*+2)^2^)(|*x*|/(1 + |*x*|)) + 1 + (1/32)  sin^2^⁡*y*  , and *h*_2_(*t*, *x*, *y*) = (1/32*π*)sin⁡(2*πx*) + |*y*|/16(1 + |*y*|) + 1/2. Note that

(24) $$\begin{array}{c} \left|h_{1}\left(t,x_{2},y_{2}\right)-h_{1}\left(t,x_{1},y_{1}\right)\right|\leq \frac{1}{16}\left|x_{2}-x_{1}\right|+\frac{1}{16}\left|y_{2}-y_{1}\right|,\\ \left|h_{2}\left(t,x_{2},y_{2}\right)-h_{2}\left(t,x_{1},y_{1}\right)\right|\leq \frac{1}{16}\left|x_{2}-x_{1}\right|+\frac{1}{16}\left|y_{2}-y_{1}\right|,\\ \nu_{1}\left(\eta_{1}+\eta_{2}\right)+\nu_{2}\left(\zeta_{1}+\zeta_{2}\right)=\frac{2}{3\sqrt{\pi }}\approx 0.3762217<1. \end{array}$$

Thus all the conditions of Theorem 4 are satisfied and, consequently, there exists a unique solution for the problem (23) on [0,1].

In our second result, we discuss the existence of solutions for the problem (1) by means of Leray-Schauder alternative.

**Lemma 6 (Leray-Schauder alternative [38, page 4]).**

Let *F* : *G* → *G* be a completely continuous operator (i.e., a map that is restricted to any bounded set in *G* is compact). Let *P*(*F*) = {*x* ∈ *G* : *x* = *λFx* *forsome* 0 < *λ* < 1}. Then either the set *P*(*F*) is unbounded or *F* has at least one fixed point.

**Theorem 7 .**

Assume that conditions (*A*_1_) and (*A*_2_) hold. Furthermore, it is assumed that *ν*_1_ *ρ*_1_ + *ν*_2_ *σ*_1_ < 1 and *ν*_1_ *ρ*_2_ + *ν*_2_ *σ*_2_ < 1, where *ν*_1_ and *ν*_2_ are given by (12). Then the boundary value problem (1) has at least one solution.

**Proof**

We will show that the operator Θ : *U* × *V* → *U* × *V* satisfies all the assumptions of Lemma 6. In the first step, we prove that the operator Θ is completely continuous. Clearly, it follows by the continuity of functions *f*_1_, *f*_2_, *h*_1_, and *h*_2_ that the operator Θ is continuous.

Let *M* ⊂ *U* × *V* be bounded. Then we can find positive constants *N*_1_ and *N*_2_ such that

(25) |h1(t,x(t),y(t))|≤N1,  |h2(t,x(t),y(t))|≤N2,  ∀(x,y)∈M.

Thus for any *x*, *y* ∈ *M*, we can get

(26) |Θ1(x,y)(t)| ≤μf1{∫0t(t−s)δ−1Γ(δ)|h1(s,x(s),y(s))|ds  +∫01(1−s)δ−1Γ(δ)|h1(s,x(s),y(s))|ds} ≤μf1N12Γ(δ+1),

which yields

(27) $$\begin{array}{c} ||\Theta_{1}\left(x,y\right)||\leq \frac{2\mu_{f_{1}}N_{1}}{\Gamma \left(\delta +1\right)}=N_{1}\nu_{1}. \end{array}$$

In a similar manner, one can show that

(28) $$\begin{array}{c} ||\Theta_{2}\left(x,y\right)||\leq \frac{2\mu_{f_{2}}N_{2}}{\Gamma \left(\omega +1\right)}=N_{2}\nu_{2}. \end{array}$$

From the inequalities (27) and (28), we deduce that the operator Θ is uniformly bounded.

Now we show that the operator Θ is equicontinuous. For that, we take *τ*_1_, *τ*_2_ ∈ [0,1] with *τ*_1_ < *τ*_2_ and obtain

(29) |Θ1(x(τ2),y(τ2))−Θ1(x(τ1),y(τ1))| ≤μf1N1|∫0τ1(τ1−s)δ−1Γ(δ)ds−∫0τ2(τ2−s)δ−1Γ(δ)ds|  +μf1N1|τ2−τ1|∫01(1−s)δ−1Γ(δ)ds =μf1N1|∫0τ1(τ1−s)δ−1−(τ2−s)δ−1Γ(δ)ds  −∫τ1τ2(τ2−s)δ−1Γ(δ)ds|  +μf1N1|τ2−τ1|∫01(1−s)δ−1Γ(δ)ds⟶0,|Θ2(x(τ2),y(τ2))−Θ2(x(τ1),y(τ1))| ≤μf2N2|∫0τ1(τ1−s)ω−1−(τ2−s)ω−1Γ(ω)ds−∫τ1τ2(τ2−s)ω−1Γ(ω)ds|  +μf2N2|τ2−τ1|∫01(1−s)ω−1Γ(ω)ds,

which tend to 0 independently of (*x*, *y*). This implies that the operator Θ(*x*, *y*) is equicontinuous. Thus, by the above findings, the operator Θ(*x*, *y*) is completely continuous.

In the next step, it will be established that the set *P* = {(*x*, *y*) ∈ *U* × *V*∣(*x*, *y*) = *λ*Θ(*x*, *y*), 0 ≤ *λ* ≤ 1} is bounded. Let (*x*, *y*) ∈ *P*; then we have (*x*, *y*) = *λ*Θ(*x*, *y*). Thus, for any *t* ∈ [0,1], we can write

(30) $$\begin{array}{c} x\left(t\right)=\lambda \Theta_{1}\left(x,y\right)\left(t\right),\;\;y\left(t\right)=\lambda \Theta_{2}\left(x,y\right)\left(t\right). \end{array}$$

Then

(31) $$\begin{array}{c} \left|x\left(t\right)\right|\leq \frac{2\mu_{f_{1}}}{\Gamma \left(\delta +1\right)}\left(\rho_{0}+\rho_{1}||x||+\rho_{2}||y||\right),\\ \left|y\left(t\right)\right|\leq \frac{2\mu_{f_{2}}}{\Gamma \left(\omega +1\right)}\left(\sigma_{0}+\sigma_{1}||x||+\sigma_{2}||y||\right), \end{array}$$

which imply that

(32) $$\begin{array}{c} ||x||\leq \nu_{1}\left(\rho_{0}+\rho_{1}||x||+\rho_{2}||y||\right),\\ ||y||\leq \nu_{2}\left(\sigma_{0}+\sigma_{1}||x||+\sigma_{2}||y||\right). \end{array}$$

In consequence, we have

(33) ||x||+||y||=(ν1ρ0+ν2σ0)+(ν1ρ1+ν2σ1)||x||+(ν1ρ2+ν2σ2)||y||,

which, in view of (13), can be expressed as

(34) $$\begin{array}{c} ||\left(x,y\right)||\leq \frac{\nu_{1}\rho_{0}+\nu_{2}\sigma_{0}}{\nu_{0}}. \end{array}$$

This shows that the set *P* is bounded. Hence all the conditions of Lemma 6 are satisfied and consequently the operator Θ has at least one fixed point, which corresponds to a solution of the problem (1). This completes the proof.
